# High thermal tolerance in high‐elevation species and laboratory‐reared colonies of tropical bumble bees

**DOI:** 10.1002/ece3.9560

**Published:** 2022-12-04

**Authors:** Victor H. Gonzalez, Kennan Oyen, Marlene L. Aguilar, Andres Herrera, Ruben D. Martin, Rodulfo Ospina

**Affiliations:** ^1^ Undergraduate Biology Program and Department of Ecology and Evolutionary Biology University of Kansas Lawrence Kansas USA; ^2^ Department of Biological Sciences, McMicken College of Arts and Sciences University of Cincinnati Cincinnati Ohio USA; ^3^ Universidad Militar Nueva Granada Cajicá Colombia; ^4^ Laboratorio de Investigaciones en Abejas Universidad Nacional de Colombia Santa Fé de Bogotá Colombia

**Keywords:** Andes, Colombia, pollinators, upper and lower thermal limits

## Abstract

Bumble bees are key pollinators with some species reared in captivity at a commercial scale, but with significant evidence of population declines and with alarming predictions of substantial impacts under climate change scenarios. While studies on the thermal biology of temperate bumble bees are still limited, they are entirely absent from the tropics where the effects of climate change are expected to be greater. Herein, we test whether bees' thermal tolerance decreases with elevation and whether the stable optimal conditions used in laboratory‐reared colonies reduces their thermal tolerance. We assessed changes in the lower (CT_Min_) and upper (CT_Max_) critical thermal limits of four species at two elevations (2600 and 3600 m) in the Colombian Andes, examined the effect of body size, and evaluated the thermal tolerance of wild‐caught and laboratory‐reared individuals of *Bombus pauloensis*. We also compiled information on bumble bees' thermal limits and assessed potential predictors for broadscale patterns of variation. We found that CT_Min_ decreased with increasing elevation, while CT_Max_ was similar between elevations. CT_Max_ was slightly higher (0.84°C) in laboratory‐reared than in wild‐caught bees while CT_Min_ was similar, and CT_Min_ decreased with increasing body size while CT_Max_ did not. Latitude is a good predictor for CT_Min_ while annual mean temperature, maximum and minimum temperatures of the warmest and coldest months are good predictors for both CT_Min_ and CT_Max_. The stronger response in CT_Min_ with increasing elevation, and similar CT_Max_, supports Brett's heat‐invariant hypothesis, which has been documented in other taxa. Andean bumble bees appear to be about as heat tolerant as those from temperate areas, suggesting that other aspects besides temperature (e.g., water balance) might be more determinant environmental factors for these species. Laboratory‐reared colonies are adequate surrogates for addressing questions on thermal tolerance and global warming impacts.

## Graphical abstract

We assessed the thermal tolerance (upper and lower) of bumble bees, a major group of pollinators that are considered at risk globally by changes in landscape and climate.

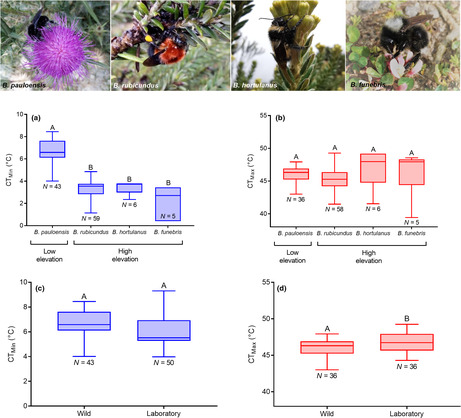

## 1 INTRODUCTION

Bumble bees (genus *Bombus* Latreille) are a group of about 280 species of eusocial bees that are widely recognized as key pollinators of wild and cultivated plants, with some species managed and commercially available for crop pollination in several countries (Ascher & Pickering, 2022; Velthuis & Van Doorn, 2006). However, bumble bees are also among the few bees with documented significant changes in population vigor and geographical range shifts due to various anthropogenic factors, including climate change (Bommarco et al., 2012; Cameron et al., 2011; Colla & Packer, 2008; Martins & Melo, 2010; Soroye et al., 2020; Williams & Jepsen, 2014). In addition, studies under climate change scenarios using niche modeling approaches suggest significant reductions (up to 78%) in bumble bees' climatically suitable areas across North and South America, Europe, and East Asia (da Silva Krechemer & Marchioro, 2020; Françoso et al., 2019; Martínez‐López et al., 2021; Naeem et al., 2019). Furthermore, species richness of bumble bees is also expected to be reduced by changes in climatic conditions (Sirois‐Delisle & Kerr, 2018; Soroye et al., 2020). Thus, current bumble bee population trends and predictions under climate change scenarios will likely influence agriculture, pollination services, and global economies.

While bumble bees are species rich and abundant in temperate areas, only a few species inhabit the tropics (Michener, 2007). For example, 50 species occur in the United States, whereas only nine species are in Colombia, a South American country located near the Equator (Ascher & Pickering, 2022; Liévano et al., 1991). Although most Colombian bumble bees are rare in comparison with other tropical bees (e.g., stingless bees) and are primarily restricted to mid‐ and high elevations in the Andes (Gonzalez & Engel, 2004; Liévano et al., 1991), their role as pollinators appears to be significant. In Colombia, the highest plant diversity and most agricultural crops are in the Andean region (Gonzalez & Engel, 2004; Rangel‐Ch, 2015), and bumble bees are among the few bee species that are present year‐round due to their perennial nests with oscillating monogyny and polygyny (one or multiple active queens) colony cycles (Gonzalez et al., 2004). In addition, bumble bees visit a wide range of plants (Pinilla‐Gallego & Nates‐Parra, 2015; Riaño‐Jiménez et al., 2020) and can fly at low temperatures, when other bees are unable to do so, because of their large body and thermoregulation capabilities (Bishop & Armbruster, 1999; Heinrich, 1979). Unfortunately, tropical bumble bees are expected to be more vulnerable to climate change than those from other latitudes, as tropical organisms appear to be living close to their maximum tolerable temperature and may have limited acclimation capacities (Deutsch et al., 2008; Kingsolver et al., 2013). However, information on tropical organisms including bumble bees is limited, and the implications of this pattern for their vulnerability to climate change remain poorly investigated (Kellermann & van Heerwaarden, 2019). Assessing organisms' responses to temperature is important because temperature influences many aspects of life, from metabolic rates to activity patterns (Huey & Kingsolver, 1989; Retana & Cerdá, 2000). In addition, thermal tolerance determine species' fundamental niche and thus have a strong influence on the species' potential distribution (Angilletta, 2009; Sunday et al., 2011). Therefore, in this study, we were interested in assessing the thermal tolerance of Colombian bumble bees by estimating their Critical Thermal Limits, the minimum (CT_Min_) and maximum (CT_Max_) temperatures at which an animal can maintain muscle control (Lutterschmidt & Hutchison, 1997).

Thermal limits are physiological traits measured under controlled conditions in the laboratory when organisms are exposed to constant (static protocols) or increasing or decreasing temperatures (dynamic protocols), and they help to explain their potential response to extreme temperature changes (Gonzalez, Oyen, et al., 2022; Roeder et al., 2021). However, these estimates may vary in response to a myriad of factors including life history traits (Baudier et al., 2018; Hamblin et al., 2017), abiotic conditions (Bujan et al., 2020; Roeder et al., 2021), environmental stressors (Gonzalez, Hranitz, et al., 2022; González‐Tokman et al., 2021), and experimental conditions (Gonzalez, Oyen, et al., 2022; Terblanche et al., 2007). For example, some studies indicate that heat tolerance may decrease with increasing elevation (García‐Robledo et al., 2016; Gonzalez et al., 2020), increase with increasing body size (Baudier et al., 2018; Oyen et al., 2016), and decrease with increasing age and length of starvation (Chidawanyika et al., 2017; Nyamukondiwa & Terblanche, 2009). Therefore, we were also interested in determining the effect of elevation and body size in tropical bumble bees' thermal limits, as well as the effect of stable optimal conditions used in laboratory‐reared colonies, which are increasingly used for research purposes. Such data will improve predictions of tropical bumble bees' response to climate change and will test whether laboratory‐reared colonies can be appropriate for addressing questions on thermal tolerance and global warming impacts.

Herein, we use the climate variability hypothesis (CVH) as a theoretical framework to test whether thermal tolerance decreases with elevation, and if the stable optimal laboratory conditions will reduce bees' thermal tolerance. The CVH asserts that organisms living in environments with great variation in temperature have a broader range of thermal tolerance than those living in more constant environments (Janzen, 1967). Thus, because the mean annual air temperature decreases linearly with altitude (~6.5°C for 1 km in elevation) (Dillon et al., 2006) but does not affect temperature variation (Baudier et al., 2018), we predict that species or populations of the same species living at high elevations would display both lower CT_Max_ and CT_Min_ than low‐elevation populations or species, but their thermal tolerance breadth (TB) (difference between CT_Max_ and CT_Min_) will be similar. Given that bees from wild colonies experience daily and seasonally fluctuating temperatures, we predict they will display higher thermal tolerance (high CT_Max_ and low CT_Min_) and thermal breadth than bees from laboratory‐reared colonies. Finally, we compiled from the literature critical thermal data for other species of bumble bees and assessed how they relate to latitude and climate variables.

## 2 MATERIALS AND METHODS

### 2.1 Bee species and study locations

We conducted thermal limits assays with four of the nine species of bumble bees that occur in Colombia: *Bombus* (*Cullumanobombus*) *hortulanus* Friese, *B*. (*Cullumanobombus*) *funebris* Smith, *B*. (*Thoracobombus*) *pauloensis* Friese, and *B*. (*Cullumanobombus*) *rubicundus* Skorikov. All species are restricted to mid‐ and high elevations across the Andean region, from Venezuela to northern Chile, except for *B. pauloensis* that also occurs in Argentina, Brazil, Paraguay, and Uruguay, from warm, low‐land tropical and subtropical environments to cold, high‐altitude ecosystems (Liévano et al., 1991; Moure & Melo, 2012). Among these species, *B. funebris* reaches the highest elevations in the Andes, as it has been recorded at 4750 m in Colombia (Gonzalez & Engel, 2004). Information on the biology of these bumble bees is limited, except for *B. pauloensis* that has been more intensively studied in Brazil. However, during the last two decades, *B. pauloensis* has been studied in Colombia for its promising use in greenhouse tomato pollination, but it is only raised in captivity at commercial scale in Argentina (Gonzalez et al., 2004; Pinilla‐Gallego et al., 2016). *Bombus pauloensis*, and to some degree *B. hortulanus*, is typically associated with transformed habitats in Colombia while the other two species are primarily found in areas with more preserved vegetation (Gonzalez et al., 2004; Pinilla‐Gallego et al., 2016).

Between February and May 2021, we collected bumble bees from two locations in the Department of Cundinamarca, Colombia, chosen for their accessibility, abundance of bumble bees, and range of elevations: Tenjo (4°51.410′N, 74°06.468′W, 2589 m), an agricultural area on the Bogota's high plain, and Matarredonda (4°33.121′N, 73°59.927′W, 3400–3600 m), an area with preserved Páramo vegetation about 40 km southeast of Tenjo (Figure S1). The composition and abundance of bumble bees varied between sites, with *B. pauloensis* occurring in Tenjo while *B. hortulanus*, *B. funebris*, and *B. rubicundus* in Matarredonda. At each location, we collected bees with a net and transferred them individually to plastic containers, which we then capped with fabric (1 mm mesh). We fed bees ad libitum with a drop of 1.5 M sucrose solution placed at the bottom of the vial. We transported them to the laboratory within 2 h of collection inside an empty Styrofoam cooler for subsequent bioassays. At each location, we measured ambient temperature and humidity using iButton data loggers (DS1923 Hygrochron™; Maxim Integrated), which we protected from solar radiation with aluminum foil and hung at 1 m above ground from tree branches. We set up two data loggers five meters apart at each location and recorded temperature and humidity every 30 min for 3 consecutive days. To increase sample size, we complemented collections of *B. rubicundus* and *B. funebris* from San Cayetano (5°13.2406′N, 74°1.391′W, 3600 m), a strip of preserved Páramo about 40 km north of Tenjo. Because we did not find significant differences in the thermal limits between bees from Matarredonda and San Cayetano after accounting for body size (ANCOVA, CT_Min_, Wald *χ* ^2^ = 3.1, *df* = 1, *p* = .08; CT_Max_, *χ* ^2^ = 1.1, *df* = 1, *p* = .31; Figure S2), we combined them in the analyses.

### 2.2 Laboratory‐reared colonies of *Bombus pauloensis*

To assess for differences in the thermal limits between wild‐caught and laboratory‐reared bees, we tested individuals from colonies of *B. pauloensis* that were initiated from gynes captured in Sopó, Cundinamarca (4°55′N, 73°56′W, 2600 m). We captured bees from Sopó, about 20 km east of Tenjo, because of the abundance of gynes, proximity to the laboratory, and similar climate, elevation, and vegetation to Tenjo, where we captured wild bees for comparison. Using a net, we captured gynes in late November and early December 2020 when gynes and males are flying (Gonzalez et al., 2004) and transferred them individually to glass vials capped with fabric inside a Styrofoam cooler with an ice pack. Following the protocol described by Cruz et al. (2008), once in the laboratory at the Universidad Militar Nueva Granada, Cajicá, Colombia (4°56′N, 74°00′W, 2600 m), we placed gynes inside wooden boxes (12.5 × 7 × 5 cm) that were labeled with a unique identifier and collection date. We fed bees ad libitum with a 50% sugar solution and pollen from local honey bees and kept them in the dark inside a climatized room at a constant temperature of 23–25°C and relative humidity of 53%–84%. Gynes initiated oviposition between one and two weeks after capture and, once colonies had a small number of workers (8–10), we transferred them to a larger wooden box (12 × 19 × 18 cm) where they remained until mid‐March 2021 when we conducted our thermal experiments. We followed brood development and colony size weekly and tested 5–9 adult workers (and males, if present) from six colonies, each containing between 30 and 50 workers at the time of the experiment. Young bumble bee workers may display low CT_Max_ (Oyen & Dillon, 2018) and foragers are typically older workers within the colony. Thus, to control for potential differences of age between wild‐caught and laboratory‐reared bees, we tested bees that were at least 2 weeks old from our colonies. Because we kept colonies under the dark at constant temperature and relative humidity during the duration of the experiment, laboratory‐reared bees were never exposed to the variable daily environmental conditions experienced by those individuals from wild colonies.

### 2.3 Critical thermal limit assays

We measured heat and cold tolerances using a dynamic (ramping temperature) protocol with the Elara 2.0 (IoTherm), a portable fully programmable heating/cooling anodized aluminum stage designed for precision temperature control of laboratory and field samples. The stage was modified with a Styrofoam cooler and clear acrylic lid to minimize the impact of airflow across the aluminum sample stage and maintain temperature stability across all vials. We placed bees individually inside glass vials (50 × 15 mm, 3.70 cm^3^) and plugged them with a moistened cotton ball (~0.2 ml of distilled water per cotton ball) to ensure enough humidity during the assays. We used an initial temperature of 22°C and held bees for 10 min at this temperature before increasing it or decreasing it at a rate of 0.5°C min^−1^. The rate of temperature change used in dynamic assays influences thermal tolerance, with studies demonstrating differential responses among species and traits, ranging from an increase or decrease in one or both thermal limits to no response (e.g., Chown et al., 2009; Oyen & Dillon, 2018; Terblanche et al., 2007). Thus, in our assays, we chose a rate of temperature change of 0.5°C min^−1^, which is an intermediate value among those reported in the literature in studies exploring insects' thermal limits, including bees (e.g., García‐Robledo et al., 2016, 2018; Gonzalez, Oyen, et al., 2022; Oyen et al., 2016). This intermediate ramping rate also reduces the time required for each experiment and minimizes the effect of confounding physiological stressors, such as dehydration or starvation. We placed vials horizontally on the stage to avoid bees from climbing along the vial. To estimate the temperature inside the vials, we placed a K‐type thermocouple inside two empty glass vials plugged with a cotton ball. We individually tracked these vial temperatures using a TC‐08 thermocouple data logger (Pico Technology). As an approximation of bees' thermal limits, we used the temperature at which bees show signs of curling (CT_Min_, Oyen & Dillon, 2018) or lost muscular control, spontaneously flipping over onto their dorsa and spasming (CT_Max_, García‐Robledo et al., 2016, 2018; Lutterschmidt & Hutchison, 1997). Then, for each specimen, we recorded its minimum intertegular distance (ITD) as a proxy of body size (Cane, 1987). We tested the same individual for CT_Max_ and CT_Min_, starting by measuring CT_Min_ with a period of acclimation at room temperature (20 min at 20–22°C) before measuring CT_Max_. Pilot experiments indicated that bees held in similar glass vials adjacent to the Elara 2.0 at room temperature survived through the duration of the assays.

### 2.4 Intertegular distance

Body size might influence estimates of thermal limits (Oyen & Dillon, 2018). Thus, after performing thermal limit assays, we measured the ITD of each specimen using an S6E stereomicroscope with an ocular micrometer (Leica Microsystems). Voucher specimens are in the Laboratorio de Abejas of the Universidad Nacional de Colombia, Bogotá, Universidad Militar Nueva Granada, Cajicá, Colombia, and in the Division of Entomology, University of Kansas Natural History Museum (Biodiversity Institute), Lawrence, Kansas.

### 2.5 Species comparisons and environmental predictors of thermal limits

To explore potential predictors of broadscale patterns of variation in bumble bees' thermal limits, we built a dataset consisting of the thermal data for the four species we studied along with published records for other species. We included geographical coordinates for each studied population, which we used to extract 19 bioclimatic variables at 10′ resolution from the MERRAclim database (Vega et al., 2018). From these variables, we excluded the following four because they are known to contain spatial artifacts as a result of combining temperature and humidity information (Escobar et al., 2014): mean temperature of most humid quarter (BIO8), mean temperature of least humid quarter (BIO9), specific humidity mean of warmest quarter (BIO18), and specific humidity mean of coldest quarter (BIO19). We used this dataset to test for association between bees' thermal limits, latitude, and climate variables, as well as to assess the relative contribution of each predictor variable on bees' thermal tolerance (see Section 2.6 below).

### 2.6 Data analyses

We conducted statistical analyses in R (R Core Team, 2018). To test for differences in the daily air temperatures and relativity humidity between locations, we used a linear mixed‐effect model (LMM) with the lmer function in the lme4 package. In this model, we used location as fixed factor and sensor identity as a random factor. To test for differences in body size (ITD) among species and between sexes (males and workers), we implemented a linear model using the lm function (Bates et al., 2015) with species and sex as fixed factors. To evaluate the relationship between body size and CT_Min_ and CT_Max_, we implemented a linear regression analysis using the lm function. We used an ANCOVA to compare CT_Min_, CT_Max_, and thermal breadth (CT_Max_ − CT_Min_) between species and sexes while controlling for the effects of body size. We used the lm function to fit a linear model and used species and sex as fixed factors and ITD as covariate. To compare CT_Min_, CT_Max_, and thermal breadth between wild‐caught and laboratory‐reared bees, we used a mixed‐model ANCOVA by implementing an LMM. In this model, we used species and sex as fixed factors, ITD as covariate, and colony identity as a random factor. We assessed the significance of fixed effects using a Type II Wald *χ* ^2^ test with the car package (Fox & Weisberg, 2011). When factors and factor interactions were significant, we used the lsmeans package (Lenth, 2016) to conduct multiple pairwise comparisons with Bonferroni adjustment to assess for differences among groups. To test for association between bees' thermal limits, latitude, and climate variables, we first implemented a linear model with either CT_Min_ or CT_Max_ as the response variable and latitude and climate variable as a predictors. Then, we used the function stepAIC from the MASS package (Venables & Ripley, 2002) to select the model with the fewest predictors based on the Akaike information criterion (AIC) using both forward and backward predictor selection. We assessed the relative importance of each predictor with the function calc.relimp from the relaimpo package (Gröemping, 2007).

## 3 RESULTS

### 3.1 Ambient temperature and humidity

Temperature and relative humidity differed significantly between locations. The mean hourly air temperature in the low‐elevation site (Tenjo) was 14.34°C (6.13–24.19°C ± 0.483, *N* = 146), whereas that of the high‐elevation site (Matarredonda) was 9.82°C (5.19–16.25°C ± 0.21, *N* = 190), and such a difference was significant (Wald *χ* ^2^ = 96.21, *df* = 1, *p* < .001). Mean hourly air relative humidity was lower in the low‐elevation site (80.67%, 33.39%–100% ± 20.06, *N* = 146) than in the high‐elevation site (90.29%, 55.46%–100% ± 9.85, *N* = 190), and that difference was also significant (Wald *χ* ^2^ = 31.00, *df* = 1, *p* < .001).

### 3.2 Critical thermal limits and body size

**FIGURE 1:**
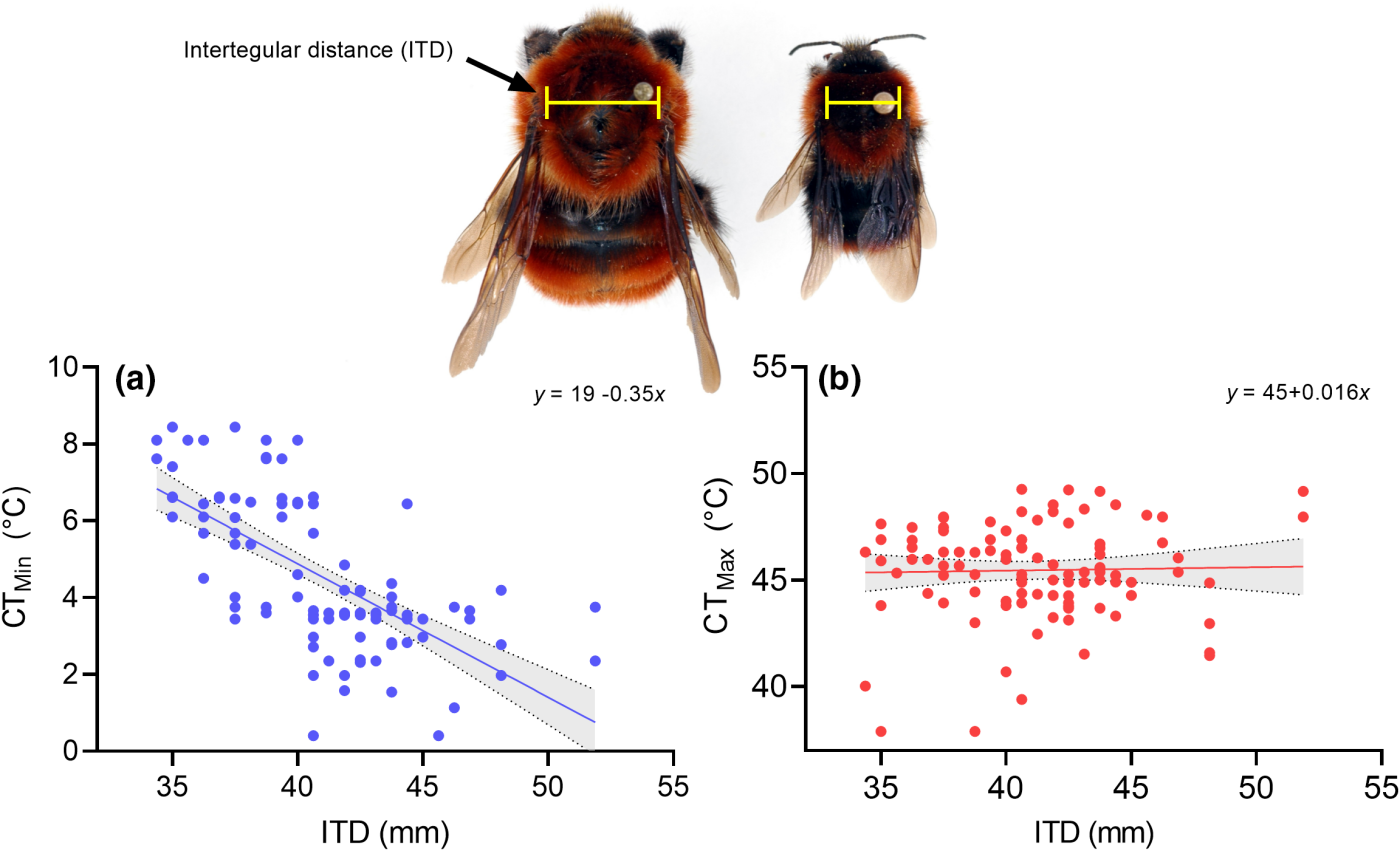
Relationship between intertegular distance (ITD) and critical thermal minima (CT_Min_) (a) and maxima (CT_Max_) (b) across all four species of bumble bees captured at two elevations in the Colombian Andes.

Intertegular distance varied significantly among species (Table 1; Wald *χ* ^2^ = 676.84, *df* = 3, *p* < .001) and between sexes (*χ* ^2^ = 74.10, *df* = 1, *p* < .001). The interaction between species and sex was not significant (*χ* ^2^ = 13.53, *df* = 3, *p* = .48). Pairwise comparisons with Bonferroni adjustment revealed that collected specimens of *B. pauloensis* were smaller than those of the other three species, which did not differ among each other (Table S1). Males were larger than workers (*p* < .001). Across all species, as well as in workers and males, CT_Min_ decreased significantly with increasing ITD (Figure 1a; All species: *p* < .001, *R* ^2^ = −0.35 ± 0.04; worker: *p* < .001, *R* ^2^ = −0.40 ± 0.04; males: *p* = .02, *R* ^2^ = −0.27 ± 0.10). Within species, CT_Min_ decreased significantly with increasing ITD only for *B. rubicundus* (*p* = .04, *R* ^2^ = −0.08 ± 0.04), but did not change with increasing ITD for remaining species. In contrast, CT_Max_ did not increase significantly with increasing ITD across all species (Figure 1b; *p* = .88, *R* ^2^ = 0.02 ± 0.06).

**TABLE 1:** Intertegular distance (ITD) and critical thermal minima (CT_Min_) and maxima (CT_Max_) of workers (♀), males (♂), and gynes (☿) of bumble bees in the Colombian Andes.

Species	Sex (*N*)	ITD (mm)	CT_Min_ (°C)	CT_Max_ (°C)
Low elevation (Tenjo)				
*Bombus pauloensis*	♀ (40)	37.69 ± 0.34	6.67 ± 0.16	45.27 ± 0.41
	♂ (3)	39.38 ± 0.96	6.32 ± 0.12	46.01 ± 1.03
High elevation (Matarredonda)				
*Bombus funebris*	♀ (4)	41.09 ± 0.30	2.32 ± 0.7	46.00 ± 2.20
	♂ (2)	46.25 ± 0.63	1.92 ± 1.52	47.05 ± 1.00
*Bombus hortulanus*	♀ (1)	43.75 ± 0.00	3.76 ± 0.00	49.17 ± 0.00
	♂ (4)	48.28 ± 2.17	3.37 ± 0.34	46.66 ± 1.73
*Bombus rubicundus*	♀ (57)	42.46 ± 0.32	3.34 ± 0.10	45.24 ± 0.22
	♂ (2)	46.25 ± 1.88	3.11 ± 0.33	42.46 ± 0.86
	☿ (2)	58.44 ± 0.31	0.52 ± 0.15	45.24 ± 0.14

*Note*: Average ± SE.

### 3.3 Critical thermal limits and elevation

**FIGURE 2:**
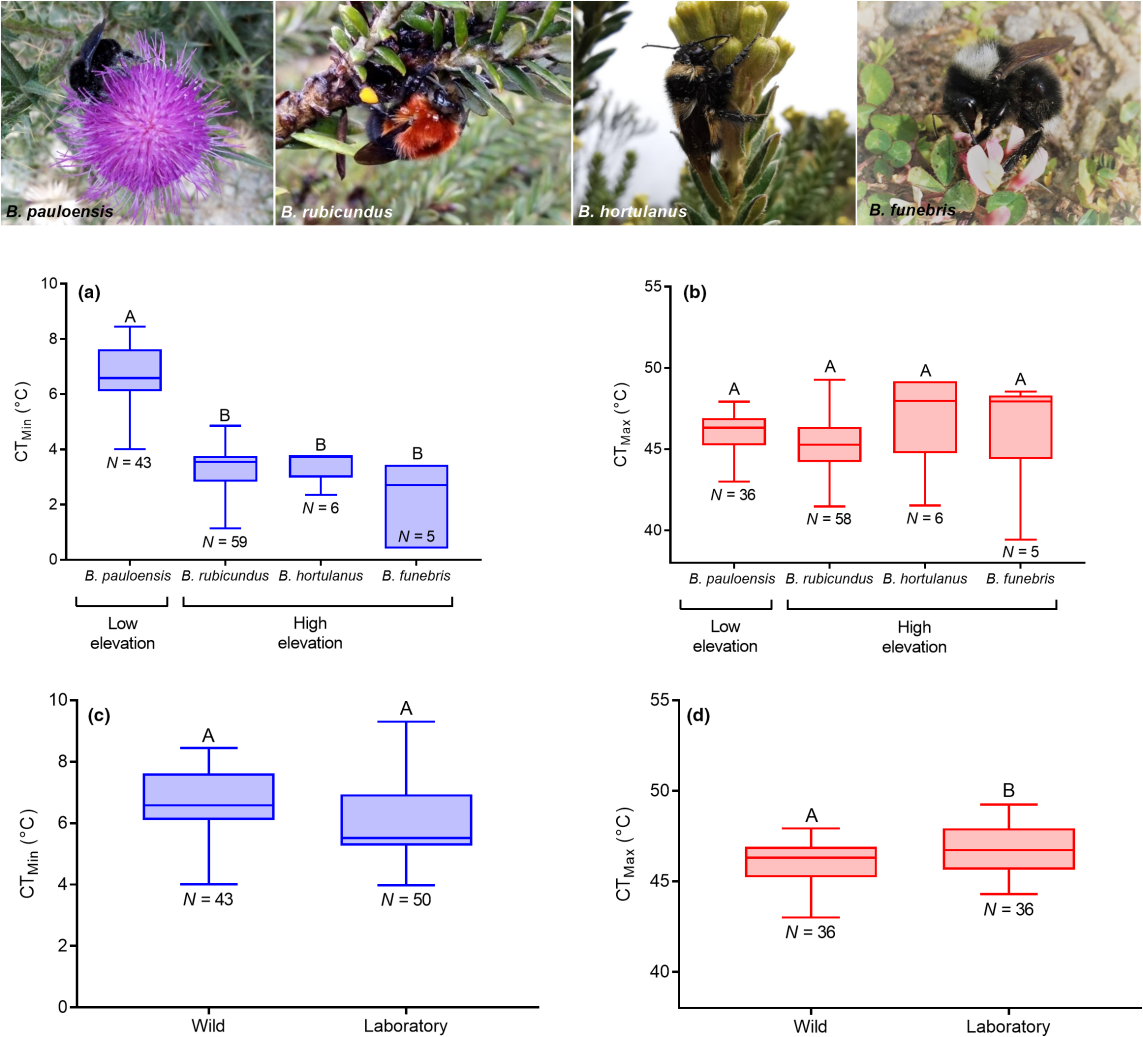
Box plots showing critical thermal minima (CT_Min_) and maxima (CT_Max_) among species of bumble bees from two elevations in the Colombian Andes (a, b). Comparison of the thermal limits between wild‐caught and laboratory‐raised individuals of *Bombus pauloensis* (c, d). For each thermal limit, groups with different letters above bars are significantly different (*p* < .05).

CT_Min_ ranged from as low as 2.19°C in *B. funebris* to as high as 6.65°C in *B. pauloensis*, and such a difference in CT_Min_ among species was significant after accounting for body size (ANCOVA, Wald *χ* ^2^ = 134.5, *df* = 3, *p* < .001). Pairwise comparisons with Bonferroni adjustment revealed that *B. pauloensis* was less cold tolerant than any of the other three species, which did not differ significantly in their CT_Min_. On average, the CT_Min_ of the three high‐elevation species was between 3.2 and 4.5°C lower than that of *B. pauloensis* (Table 1, Figure 2a). CT_Min_ was similar between sexes (*χ* ^2^ = 3.21, *df* = 2, *p* = .13). After accounting for body size, CT_Max_ (Figure 2b) was similar among all species (ANCOVA, Wald *χ* ^2^ = 24.0, *df* = 3, *p* = .18) and between sexes (*χ* ^2^ = 2.36, *df* = 2, *p* = .78). As for CT_Min_, thermal breadth differed among species after accounting for body size (Wald *χ* ^2^ = 143.3, *df* = 3, *p* < .001). It was narrower in *B. pauloensis* (39.34 ± 0.31°C vs. 42.0–44.16°C) than in the remaining species, which did not differ significantly in their thermal breadth among each other.

### 3.4 Critical thermal limits of wild‐caught and laboratory‐reared bees

Wild‐caught bees of *B. pauloensis* were larger than those tested from colonies reared in the laboratory (Wald *χ* ^2^ = 7.33, *df* = 1, *p* < .001) and males had a greater ITD than workers (*χ* ^2^ = 8.61; *df* = 1, *p* < .001; Table 2). The interaction between bees' origin (wild‐caught and laboratory‐reared) and sex was not significant (*χ* ^2^ = 0.44, *df* = 1, *p* = .51). After accounting for body size, CT_Min_ (Figure 2c) was similar between wild‐caught and laboratory‐ reared bees (ANCOVA, Wald *χ* ^2^ = 1.10, *df* = 1, *p* = .295), as well as between sexes (*χ* ^2^ = 0.96, *df* = 1, *p* = .33). However, CT_Max_ (Figure 2d) was on average 0.84°C higher in bees reared in the laboratory than those collected in field (*χ* ^2^ = 4.40, *df* = 1, *p* = .04), but similar between sexes (*χ* ^2^ = 0.44, *df* = 1, *p* = .51). As for CT_Max_, thermal breadth was greater in laboratory‐reared bees than in wild‐caught bees (40.79°C vs. 39.34°C; *χ* ^2^ = 26.13, *df* = 1, *p* = .011), but similar between sexes (*χ* ^2^ = 12.22, *df* = 1, *p* = .08).

**TABLE 2:** Intertegular distance (ITD) and critical thermal minima (CT_Min_) and maxima (CT_Max_) of workers (♀) and males (♂) of wild‐caught and laboratory‐reared bumble bees (*Bombus pauloensis*).

Origin	Sex	ITD (mm)	CT_Min_ (°C)	CT_Max_ (°C)
Wild	♀	37.69 ± 0.34, *N* = 40	6.67 ± 0.16, *N* = 40	46.02 ± 0.22, *N* = 33
	♂	39.38 ± 0.96, *N* = 3	6.32 ± 0.12, *N* = 3	46.01 ± 1.03, *N* = 3
Laboratory	♀	36.01 ± 0.50, *N* = 42	6.11 ± 0.19, *N* = 42	46.80 ± 0.26, *N* = 28
	♂	39.98 ± 0.95, *N* = 8	5.04 ± 0.32, *N* = 8	47.03 ± 0.49, *N* = 8

*Note*: Average ± SE.

### 3.5 Species comparisons and environmental predictors

**FIGURE 3:**
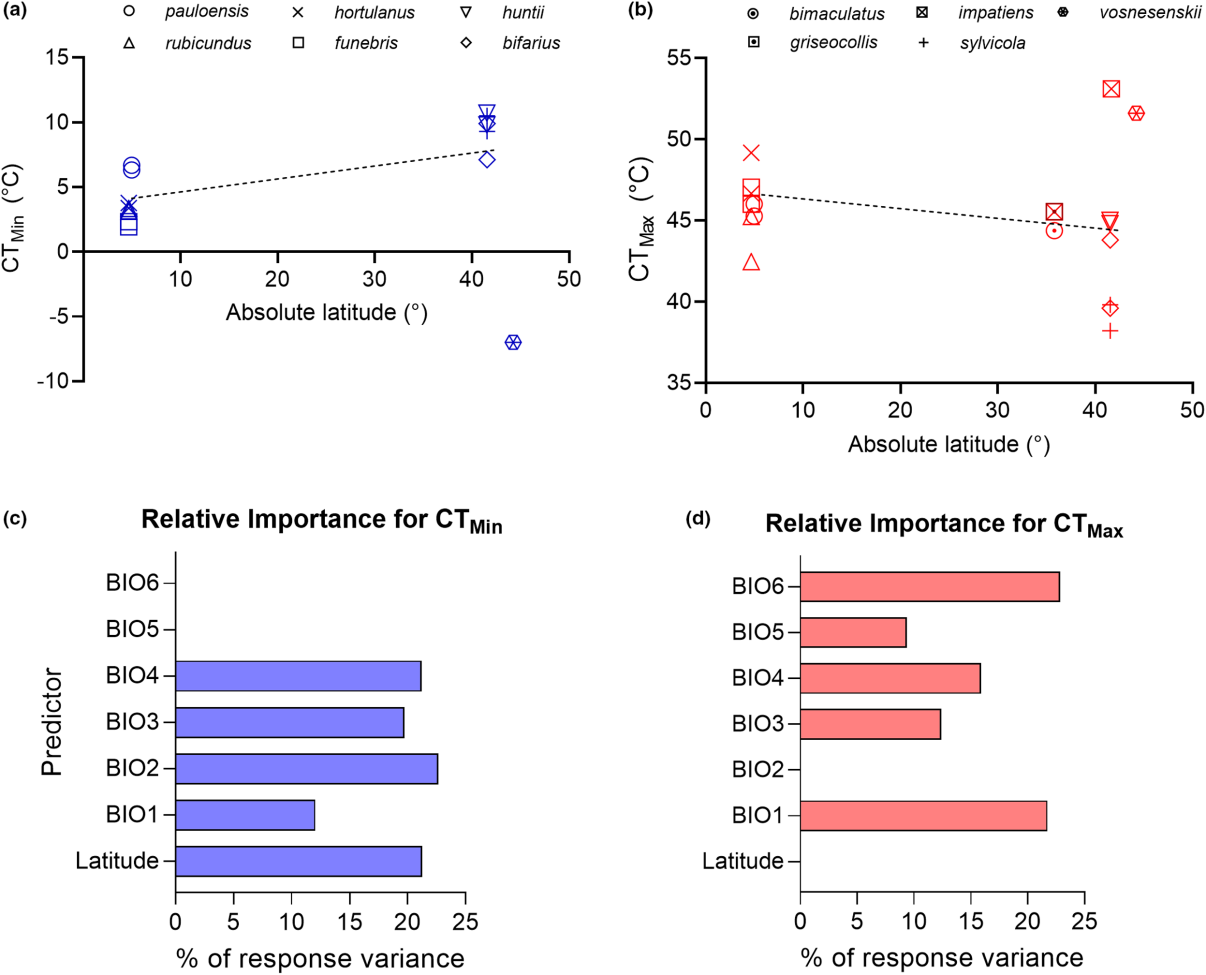
Species comparisons and environmental predictors. (a, b) Relationship between absolute latitude and average values of critical thermal minima (CT_Min_) and maxima (CT_Max_) recorded for bumble bees in the literature and this work (see Table S2 for details and references). (c, d) Relative importance of environmental predictors for the best models obtained for bumble bees' thermal limits. Bioclimatic variables taken from MERRAclim database.

Critical thermal limits have been assessed for seven North American bumble bee species (Figure 3a,b; Table S2). Estimates of CT_Min_ range from average values of −7°C in the worker of *B. vosneseskii* (Pimsler et al., 2020) to 10°C in the male of *B. huntii* (Oyen et al., 2016), whereas those for CT_Max_ range from 38.2°C in the male of *B. sylvicola* (Oyen et al., 2016) to 53.1°C in the worker of *B. impatiens* (Burdine & McCluney, 2019). Using Akaike's information criterion, the best model for the association between CT_Max_, latitude, and climate variables resulted in a model that could explain 75% of the variance in CT_Max_ and that combined annual mean temperature (BIO1), isothermality (BIO3), temperature seasonality (BIO4), maximum temperature of warmest month (BIO5), and minimum temperature of the coldest month (BIO 6). All these variables showed significant correlation with CTMax. For CT_Min_, the best model explained 95% of the variance and combined latitude, mean diurnal range temperature (BIO2), BIO1, BIO3, and BIO4. Only the latter variable was not significantly correlated with CT_Min_ (Table S3). In these best models, BIO1 displayed the lowest relative importance for the variance of CT_Min_ (12.1%) while this bioclimatic variable in combination with BIO6 accounted for about 44% of the variance of CT_Max_ (Figure 3c,d).

## 4 DISCUSSION

### 4.1 Critical thermal limits and elevation

We found that CT_Min_ decreased with increasing elevation while CT_Max_ was similar between elevations. We also found that while CT_Min_ was similar between wild‐caught and laboratory‐reared bees of *B. pauloensis*, CT_Max_ was slightly but significantly higher (0.84°C) in laboratory‐reared bees. Thermal breadth was greater in species from the high‐elevation site and in bees from laboratory‐reared colonies. Thus, these results are not consistent with our expectations based on the predictions of the climate variability hypothesis (CVH). Instead, the similarity in CT_Max_ and the stronger response we observed in CT_Min_ with increasing elevation follow a pattern observed across a wide range of vertebrates (Pintanel et al., 2019) and invertebrates (Bishop et al., 2017; Hoffmann et al., 2013), which is commonly known as Brett's Rule or Brett's heat‐invariant hypothesis (Brett, 1956).

While some studies have provided support to the variation in the geographic and landscape patterns of the thermal limits predicted by the CVH (Boyle et al., 2021; Burdine & McCluney, 2019; García‐Robledo et al., 2016, 2018; Gonzalez et al., 2020; Hamblin et al., 2017), some have yielded contradictory results. Recent works (Bennett et al., 2021; Oyen et al., 2016; Oyen & Dillon, 2018; Sunday et al., 2019) demonstrate that CT_Max_ is less variant than CT_Min_, as the latter decreases significantly across elevation and latitude, and even across an anthropogenic gradient (Sánchez‐Echeverría et al., 2019). To date, only three studies have addressed the effect of elevation on the thermal limits of bees. One of them (Pimsler et al., 2020) agrees with our results while the other two (Gonzalez et al., 2020; Oyen et al., 2016) indicate that both CT_Min_ and CT_Max_ decline with elevation. Thus, altitudinal variations in bees' thermal limits might be taxon specific.

### 4.2 Critical thermal limits, body size, and laboratory conditions

Studies assessing for differences in thermal tolerance between wild‐caught and laboratory‐reared individuals have been conducted with both vertebrates and invertebrates, but not in bumble bees despite some species already being used in thermal biology and climate change studies. The results of these studies are mixed. For example, Lyons et al. (2012) found that wild‐caught mosquitos displayed a lower CT_Min_ than laboratory‐reared individuals and no difference in CT_Max_. A similar relatively invariant CT_Max_ has been observed in wild‐caught and laboratory‐reared fruit flies (Krebs et al., 2001). By contrast, other studies have shown either higher or lower CT_Max_ in wild‐caught individuals, as in the case of domestic trout and zebrafish (Carline & Machung, 2001; Morgan et al., 2019). As noted in these works, the different responses among studies might be related to the limited potential for variation in CT_Max_ with respect CT_Min_ (Araújo et al., 2013) or to the improved conditions in the laboratory environment that include better nutrition and reduced exposure to other stressors, such as pesticides, diseases, or parasites.

Food is known to significantly influence thermal limits in some insects (Bujan & Kaspari, 2017; Chidawanyika et al., 2017; Nyamukondiwa & Terblanche, 2009), but the effect on bees' thermal limits remains to be explored. For example, ants fed with a 10% sucrose solution for 10 h displayed a CT_Max_ 5°C higher than ants fed only with water (Bujan & Kaspari, 2017). By contrast, the thermal tolerance of *B. impatiens*, as well as that of honey bees, appears to be relatively invariant to the short‐term (<24 h) ingestion of carbohydrates (Gonzalez, Oyen, et al., 2022; Oyen & Dillon, 2018). However, cold tolerance increased in *B. terrestris* when bees were fed continuously with both pollen and nectar for several days when compared to the control bees (Owen et al., 2013). Thus, it is possible that our laboratory‐reared colonies had a better nutritional state than that of the wild colonies due to the continuous supply of pollen and nectar, which might have influenced their CT_Max_. However, we cannot rule out population differences, as at least CT_Min_ is potentially driven by genetic mechanisms in bumble bees (Pimsler et al., 2020). Nevertheless, the increased in CT_Max_ was small (<1°C) and it suggests that using thermal data from laboratory‐reared colonies will provide a reasonable approximation of expected responses in the field, which is relevant because some bumble bee species are now commercially available and thus are suitable for climate change studies.

We found that across all species, CT_Min_ decreased significantly with increasing body size while CT_Max_ did not (Figure 1). Such relationship between body size and thermal limits was also evident in the two gynes of *B. rubicundus* we assessed, which were 27% larger than workers' average ITD and displayed a similar CT_Max_ but a CT_Min_ close to zero (Table 1). Within species, CT_Min_ decreased significantly with increasing ITD only for *B. rubicundus*, but sample size for *B. hortulanus* and *B. funebris* was very limited (<5 specimens each). Studies have shown that thermal tolerance increases with increasing body size within and among species in several insect groups (Baudier et al., 2018; Cerdá & Retana, 1997; González‐Tokman et al., 2021; Janowiecki et al., 2020; Oyen et al., 2016). However, this relationship is not clear for bees. While some studies (Gonzalez et al., 2020; Hamblin et al., 2017; Oyen & Dillon, 2018) indicate no effect of body size, others demonstrate an increase in both CT_Max_ and CT_Min_ with increasing body size within and among species (Oyen et al., 2016). Thus, variations in the thermal limits in relation to body size might be species specific, as demonstrated here for at least *B. pauloensis* and *B. rubicundus*. Bumble bees are good models to explore the influence of body size on thermal limits because they display as much as 10‐fold difference in body mass within a colony (Couvillon et al., 2010). If bees' foraging ability depends on both body size and their thermal limits, one can predict that larger bees might be able to forage under lower or higher temperatures than smaller bees. This has been investigated with *B. impatiens* and *B. terrestris* and no relationship between body size and foraging temperature has been found (Couvillon et al., 2010; Peat et al., 2005). However, the thermal limits of *B. impatiens* are relatively invariant to body size (Oyen & Dillon, 2018). Future studies should address if bee's foraging activity is related to their thermal limits.

### 4.3 Species comparisons and environmental predictors

Few studies have assessed the thermal limits of bumble bees, although several have explored bumble bees' thermal tolerance using other metrics, such as lethal thermal limits, supercooling point (Owen et al., 2013), chill‐coma recovery time (Oyen et al., 2021), and time before heat stupor (Martinet et al., 2015, 2021; Zambra et al., 2020). These studies on thermal limits show differential thermal sensitivity and thermal breadth among species. For example, while *B. huntii* displays relatively high CT_Min_ (9.8–10.7°C) and CT_Max_ (44.8–45.0°C) (Oyen et al., 2016), *B. vosneseskii* displays significantly low CT_Min_ (−7°C) and high CT_Max_ (51.6°C) (Pimsler et al., 2020). A regression analysis between CT_Min_ and CT_Max_ across all bumble bee species indicates that CT_Max_ significantly increases with decreasing CT_Min_ (*R* ^2^ = 0.49, *p* = .002; Figure S3). This suggests that improved performance at high temperature might also improve performance at low temperature in bumble bees, as documented for other ectotherms (e.g., von May et al., 2019). However, estimates of bumble bees' critical thermal limits are scarce and future works including additional species should corroborate this relationship between thermal traits. In addition, available estimates of bumble bees' thermal limits might be influenced by methodological approaches, such as the rate of temperature change used in dynamic assays. For example, estimates of CT_Min_ have been conducted with slow ramping rates (0.1 and 0.25°C min^−1^), which are known to significantly increase estimates of CT_Min_ in bumble bees (Oyen & Dillon, 2018) and honey bees (Gonzalez, Oyen, et al., 2022). Thus, relatively high estimates of CT_Min_, such as those recorded for *B. huntii*, might be biased.

In this study, we used the same individual to estimate both CT_Min_ and CT_Max_, which might influence the average estimate of bumble bees' CT_Max_. Prior exposure to low temperatures, as when bees are subjected to the CT_Min_ assays, might affect estimates of CT_Max_ by increasing the duration of the experiment at stressful temperatures and exposure to potential confounding stressors, such as starvation and desiccation. These additional stressors could lead to the production of heat‐shock proteins or the cumulative impacts of cellular damage, which might influence CT_Max_ (Overgaard et al., 2012; Sejerkilde et al., 2003; Terblanche et al., 2011). Although we did not test the effect of cold exposure on CT_Max_ in the species we studied, there is evidence that prior exposure to low temperatures during CT_Min_ assays, followed by a short recovery period similar to that used in this work, does not influence average estimates of CT_Max_ in *B. impatiens* and Africanized honey bees (Gonzalez, Oyen, et al., 2022). However, given that responses might be species specific, and that this has only been tested on a single bumble bee species, we cannot rule out entirely the possibility that prior cold exposure could have influenced average estimates of CT_Max_ in our experiments.

Despite the scarcity of data and differences in methodological approaches among studies, our estimates of critical thermal limits for Andean bumble bees' appear to be comparable with average estimate values recorded for temperate species (Figure 3a,b). Given that estimates of heat tolerance for Andean bumble bee species are significantly higher than the highest air temperature recorded in those Andean ecosystems (≤25°C), variations in temperature itself might not represent a serious threat to these Andean species. Changes in other aspects of climate that covariate with temperature, such as relative humidity, might also be important in predicting bees' responses to climate change, but desiccation tolerance is poorly known in bees even though some species seem to be highly sensitive to changes in humidity (Burdine & McCluney, 2019). However, displaying high tolerance at high elevations might help to withstand the sudden daily variations in temperature experienced in high Andean environments, which ranges from freezing temperatures and late frosts to high temperatures with high solar radiation, as it has been reported for Andean plants (Leon‐Garcia & Lasso, 2019).

Our exploratory analysis aimed at identifying environmental predictors of bumble bees' thermal limits suggests that latitude is a good predictor for CT_Min_ only (Figure 3a) and that annual mean temperature (BIO1), as well as maximum and minimum temperatures of the warmest (BIO5) and coldest month (BIO6) are good predictors for both CT_Min_ and CT_Max_. These results agree with other studies that show a strong response in CT_Min_ with changes in latitude and that extreme temperatures are also a significant underlying mechanism for explaining geographical patterns in thermal tolerance limits of terrestrial ectotherms (Sunday et al., 2019). However, our analysis is preliminary in nature given the dearth of information on bumble bees' thermal limits.

## 5 CONCLUSIONS AND FUTURE DIRECTIONS

The results of this study are consistent with variations in the thermal limits as predicted by the Brett's heat‐invariant hypothesis, in which stronger responses are expected in CT_Min_ than in CT_Max_ across both latitude and elevation. Extreme temperatures seem to be a significant mechanism underlying variation in thermal responses across species. Bees from laboratory‐reared colonies of *B. pauloensis* can be recommended as adequate testing subjects for bees' thermal biology studies. However, we conducted our studies in a narrow temporal window (dry season) and elevation and used bees from a small number of populations. In addition, bumble bees' thermal limits are known only from a few species, most of them in two subgenera (*Cullumanobombus* and *Pyrobombus*). Data from other clades are needed, as thermal limits might be constrained across the phylogeny (Kellermann et al., 2012). A recent study assessing the time before heat stupor across 39 species of bumble bees from major biogeographic regions documented low heat tolerance for cold‐adapted species and the highest heat tolerance for Mediterranean species (Martinet et al., 2021). It also reported no phylogenetic signal for this aspect of heat tolerance, but this was measured in males and tropical taxa were not included. Finally, low‐land populations of tropical bumble bees should be tested in future studies, in particular *B. pauloensis* and *B. transversalis* (Olivier, 1789), species that thrive in anthropogenic environments and are adapted to live in hot and wet tropical habitats, respectively.

## AUTHOR CONTRIBUTIONS

**Victor H. Gonzalez:** Conceptualization (lead); data curation (lead); formal analysis (lead); funding acquisition (lead); investigation (lead); methodology (lead); project administration (lead); resources (lead); software (lead); supervision (lead); validation (lead); visualization (lead); writing – original draft (lead); writing – review and editing (lead). **Kennan Oyen:** Conceptualization (lead); data curation (equal); formal analysis (equal); funding acquisition (lead); investigation (lead); methodology (lead); project administration (supporting); resources (equal); software (equal); supervision (equal); validation (equal); visualization (equal); writing – original draft (equal); writing – review and editing (equal). **Marlene L. Aguilar:** Conceptualization (equal); data curation (equal); formal analysis (supporting); funding acquisition (equal); investigation (equal); methodology (equal); project administration (equal); resources (equal); supervision (equal); validation (equal); visualization (equal); writing – original draft (equal); writing – review and editing (equal). **Andres Herrera:** Investigation (equal); methodology (equal); resources (equal); writing – review and editing (equal). **Ruben D. Martin:** Investigation (equal); methodology (equal); resources (equal); writing – review and editing (equal). **Rodulfo Ospina:** Conceptualization (lead); data curation (equal); formal analysis (equal); funding acquisition (lead); investigation (lead); methodology (lead); project administration (lead); resources (lead); supervision (equal); validation (equal); visualization (equal); writing – original draft (equal); writing – review and editing (equal).

## CONFLICT OF INTEREST

Authors declare no conflict of interests.

## Supporting information


Appendix S1

## Data Availability

The complete datasets used for the analyses in this study are available on Dryad: https://doi.org/10.5061/dryad.pzgmsbcqb.
