# Supplementary material for: High thermal tolerance in high‐elevation species and laboratory‐reared colonies of tropical bumble bees
Source: Ecol Evol. 2022 Dec 4;12(12):e9560. doi: 10.1002/ece3.9560 (PMC9720000; doi:10.1002/ece3.9560)

*Supplemental Materials*

**High thermal tolerance in high elevation species and laboratory-reared colonies of tropical bumble bees**

Victor H. Gonzalez^1*^, Kennan Oyen^2^, Marlene L. Aguilar^3^, Andres Herrera^1^, Ruben D. Martin^3^ and Rodulfo Ospina^4^

^1^Undergraduate Biology Program and Department of Ecology and Evolutionary Biology, University of Kansas, Lawrence, Kansas, 66045, U.S.A.

^2^Department of Biological Sciences, McMicken College of Arts and Sciences, University of Cincinnati, 318 College Drive, Cincinnati, OH 45221

^3^Universidad Militar Nueva Granada, Cajicá, Cundinamarca, Colombia

^4^Laboratorio de Investigaciones en Abejas, Universidad Nacional de Colombia, Santa Fé de Bogotá, Colombia

Victor H. Gonzalez: **0000-0002-4146-1634**

*Correspondence: Victor H. Gonzalez. Email: vhgonza@ku.edu

**Table S1.** Results of pairwise comparisons with Bonferroni adjustment of the intertegular distance among bumble bee species. Significant *P*-value in boldface.

| Comparison | *P*-value |
| --- | --- |
| *B. pauloensis* vs. *B. funebris* | **<0.001** |
| *B. pauloensis* vs. *B. hortulanus* | **<0.001** |
| *B. pauloensis* vs. *B. rubicundus* | **<0.001** |
| *B. funebris* vs. *B. hortulanus* | 0.249 |
| *B. funebris* vs. *B. rubicundus* | 1.000 |
| *B. hortulanus* vs. *B. rubicundus* | 0.471 |

**Table S2.** Compiled dataset of critical thermal minima (CT_Min_) and maxima (CT_Max_), geographical coordinates and bioclimatic variables. Thermal data and geographical coordinates were taken from literature while bioclimatic variables were taken from the MERRAclim database (Vega et al., 2018). This dataset was used in Figure 3 and in a linear model to explore potential predictors of broad-scale patterns in the variation of bumble bee’s thermal limits (see results of analysis in Table 3S).

**Table S3.** Parameter estimates for the linear model testing the relationship between critical thermal minima (CT_Min_) and maxima (CT_Max_), latitude, and bioclimatic variables from the MERRAclim database (Vega et al., 2018). Significant *P*-value in boldface.

| **Model** | **Term** | **Coefficient** | **S.E.** | **t** | ***P*-value** |
| --- | --- | --- | --- | --- | --- |
| CT_Min_ ~ latitude + BIO1+BIO2+BIO3+BIO4 | Intercept  Latitude  BIO1  BIO2  BIO3  BIO4 | -1.430e+03  1.117e+01  -2.356e+00  3.481e+00  1.690e+01  -1.080e-02 | 4.257e+02  3.693e+00  5.085e-01  7.140e-01  5.542e+00  9.169e-03 | -3.359  3.023  -4.633  4.876  3.058  -1.178 | **0.008**  **0.014**  **0.001**  **0.001**  **0.014**  0.269 |
| CT_Max_ ~ BIO1+BIO3+BIO4+BIO5+BIO6 | Intercept  BIO1  BIO3  BIO4  BIO5  BIO6 | -1.720e+02  -2.266e+00  3.161e+00  4.243e-02  5.284e-01  2.405e+00 | 4.062e+01  3.440e-01  5.634e-01  6.787e-03  1.085e-01  3.567e-01 | -4.235  -6.589  5.609  6.251  4.869  6.743 | **0.001**  **<0.001**  **<0.001**  **<0.001**  **<0.001**  **<0.001** |

**Figure S1.** Study areas in Colombia. Google Earth, earth.google.com/web/.


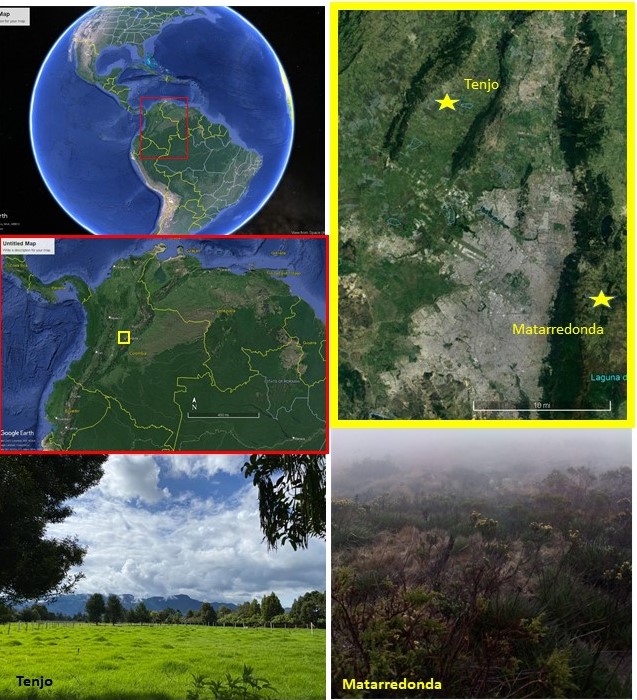


**Figure S2.** Box plots showing critical thermal minima (CT_Min_) and maxima (CT_Max_) between individuals collected in Matarredonda and San Cayetano, Cundinamarca, Colombia. San Cayetano was an additional place surveyed once to increase sample size. For each thermal limit, groups with different letters above bars are significantly different (P *<* 0.05).


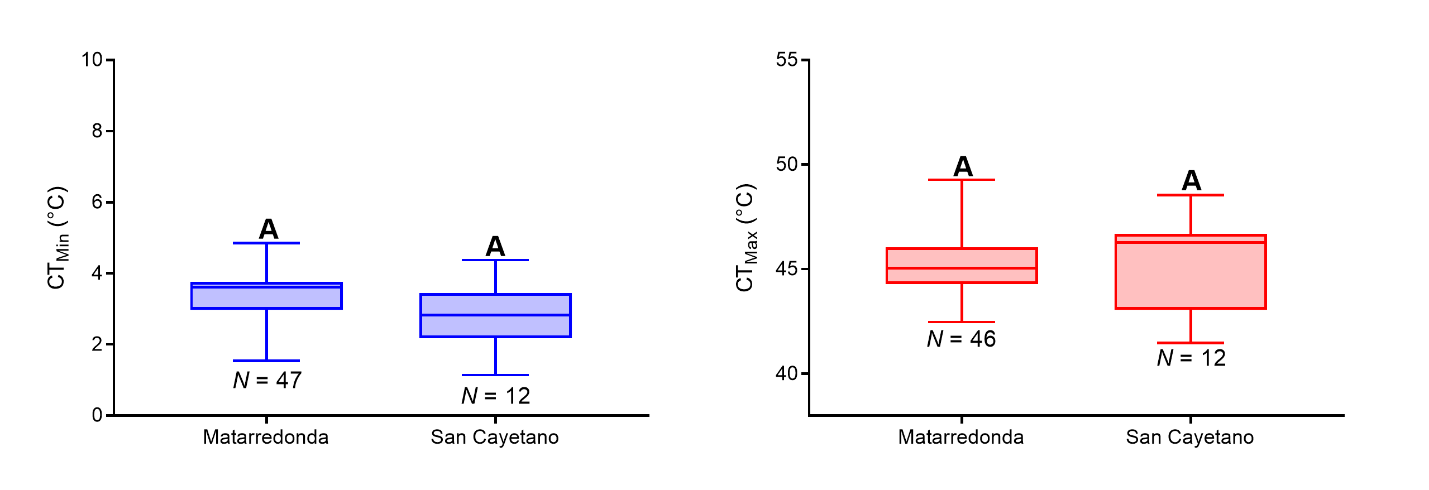


**Figure S3.** Relationship between critical thermal minima (CT_Min_) and maxima (CT_Max_) of bumble bees.


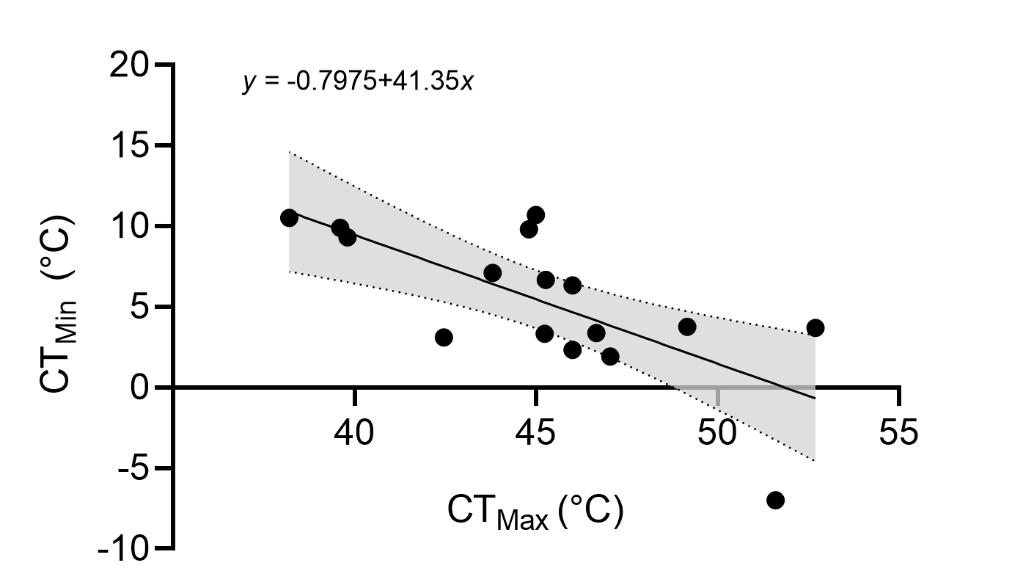

Supplement: Supplementary file 1 — Appendix S1 [file ECE3-12-e9560-s001.docx]
